# Circulating miRNAs in maternal plasma as potential biomarkers of early pregnancy in sheep

**DOI:** 10.3389/fgene.2022.929477

**Published:** 2022-08-17

**Authors:** Mustafa Hitit, Mehmet Kose, Mehmet Salih Kaya, Mesut Kırbas, Sukru Dursun, Ilyas Alak, Mehmet Osman Atli

**Affiliations:** ^1^ Department of Genetics, Faculty of Veterinary Medicine, Kastamonu University, Kastamonu, Turkey; ^2^ Department of Obstetrics and Gynecology, Faculty of Veterinary Medicine, Dicle University, Diyarbakir, Turkey; ^3^ Department of Physiology, Faculty of Medicine, Ankara Yildirim Beyazit University, Ankara, Turkey; ^4^ Bahri Dagdas International Agricultural Research Institute, Konya, Turkey; ^5^ Department of Obstetrics and Gynecology, Faculty of Veterinary Medicine, Aksaray University, Aksaray, Turkey; ^6^ Department of Animal Sciences, Vocational School of Technical Sciences, Ankara Yıldırım Beyazıt University, Ankara, Turkey; ^7^ Department of Reproduction and Artificial Insemination, Faculty of Veterinary Medicine, Harran University, Sanliurfa, Turkey

**Keywords:** ovine, plasma, microRNA, circulating, early pregnancy, expression

## Abstract

MicroRNA (miRNA) plays an important role in the control of gene expression and is implied in many biological functions, including embryo implantation and development. The aim was to assess plasma miRNA profiles during the peri-implantation and ascertain potential candidate miRNA markers for early pregnancy diagnosis in ovine plasma. The plasma samples were obtained from a total of 24 ewes on days 12 (pre-implantation; P12, *n* = 4), 16 (implantation; P16, *n* = 4) and 22 (post-implantation; P22, *n* = 4) after mating, and on their corresponding days of 12 (Pre-C; C12, *n* = 4), 16 (Imp-C; C16, *n* = 4) and 22 (Post-C; C22, *n* = 4) of the estrous cycle. The miRNA profiles in plasma were assessed by microarray technology. We detected the presence of 60 ovine-specific miRNAs in plasma samples. Of these miRNAs, 22 demonstrated a differential expression pattern, especially between the estrous cycle and early pregnancy, and targeted 521 genes. Two miRNAs (oar-miR-218a and oar-miR-1185-3p) were confirmed using RT-qPCR in the ovine plasma samples. Protein-protein interaction (PPI) network of target genes established six functional modules, of which modules 1 and 3 were enriched in the common GO terms, such as inflammatory response, defense response, and regulation of immune response. In contrast, module 2 was enriched in the developmental process involved in reproduction, embryo development, embryonic morphogenesis, and regulation of the developmental process. The results indicate that miRNAs profiles of plasma seemed to be modulated during the peri-implantation stage of pregnancy in ewes. Circulating miRNAs could be promising candidates for diagnosis in early ovine pregnancy.

## Introduction

In order to have a successful pregnancy, the uterine environment and a viable embryo must work together during the peri-implantation stage of pregnancy (Kose et al., 2016c; Kaya et al., 2017). Many molecules controlled at the gene expression level are implicated in the regulation of implantation (Kose et al., 2016a; Alak et al., 2020), but their particular regulatory mechanisms remain still unclear. Previous studies indicated that changes in microRNA (miRNA) expression play a functional role in embryo implantation in many species (Song et al., 2015; Gebremedhn et al., 2018). Apart from roles in expressed tissues of interest, particularly in the endometrium (Kiyma et al., 2015; Yan et al., 2021), miRNAs also could be released into the extracellular environment by cells, facilitating cell-cell interactions and providing valuable information related to the specific conditions (Bidarimath et al., 2021; Shi et al., 2021).

miRNAs, non-coding RNA molecules with 21–24 nucleotides, post-transcriptionally modulate gene expression and thus are implied in biological processes (Bartel, 2004; Hitit et al., 2015; WB, 2016). An understanding of miRNAs expressions and their functions in the female reproductive tract has been recently developed. Accordingly, miRNAs are functional at different stages of the reproduction process in the female (oocyte growth, maturation, embryo development, implantation, and placentation) (Guzeloglu et al., 2013; Hitit et al., 2013; Kaczmarek et al., 2020). miRNA expression profiles were shown to be different during the peri-implantation period and even differed between implantation and non-implantation regions in mice (Chakrabarty et al., 2007; Liu et al., 2015). Moreover, gene expression profiles concerning embryo implantation in ovine endometrium revealed pregnancy-associated miRNAs, of which many were differentially expressed in the endometrium on day 13 of pregnancy (Kiyma et al., 2015). Also, we determined differentially expressed miRNA profiles in the endometrium during the early pregnancy on days 12, 16, and 22 (Kose et al., 2022).

Placenta or embryo-derived miRNA molecules pass into body fluids, such as plasma, serum, and milk, within the extracellular vesicle (Tan et al., 2020). Therefore, their resistance with the stable structure to adverse effects such as freezing–thawing and high temperature is encouraging for detecting early pregnancy and monitoring the maintenance of pregnancy (De Bem et al., 2017). More specifically, in livestock animals, plasma samples from cattle and goats provide unique miRNA profiles between pregnant and non-pregnant groups, which circulating miRNAs were shown to be potential markers of early pregnancy stages and endometrial receptivity in the field of reproductive biology (Ioannidis and Donadeu, 2016; Zang et al., 2021; Zhao et al., 2021). El-Shorafa and Sharif (2016) determined that healthy fertile women exhibit different expression profiles of some miRNAs in maternal plasma than women who experienced recurrent pregnancy loss of unknown cause (El-Shorafa and Sharif, 2016). Similarly, Kotlabova et al. (2011) reported that seven miRNAs of placental origin were identified in the maternal circulation throughout pregnancy.

Considering the literature indicated above, we hypothesized that early pregnancy causes a change in plasma miRNA profiles, and this may be a candidate marker for early pregnancy diagnosis in sheep. The current study, therefore, aimed to ascertain the circulating miRNA profile in the plasma during peri-implantation, which is one of the most crucial stages for the establishment and maintenance of pregnancy in the ewe, and identify candidate miRNA markers for early pregnancy diagnosis.

## Materials and methods

### Experimental design and sample collection

All experimental steps were confirmed by the Bahri Dagdas International Agricultural Research Institute Ethical Research Committee (Number: 29/01/2016-49-7). Twenty-four multiparous ewes (*n* = 24) were used; assigned into cyclic (C, *n* = 12) and pregnant (P, *n* = 12) groups, randomly. Animal diets for 3- to 5-year-olds were adjusted to satisfy the NRC (2007) nutritional requirements. All other supplementals were given *ad libitum* during the study.

Before the experiment, cycles of the ewes were synchronized with two cloprostenol (a synthetic analog of prostaglandin F2alpha; PGF2α, 125 mcg) injections 11 days apart. Immediately after the second injection, estrus was checked three times a day using teaser rams. Estrus of ewes was obtained through teaser ram at 8 h intervals for 5 days after the second injection, and the ewes that showed estrus were recorded. Then, these ewes were followed to accomplish their entire cycle and noted for new natural estrus using teaser rams. In this new estrus, the pregnant ewes mated (day 0) two times, 12 h alone, using fertility-proven rams. The estrus day in the cyclic group was accepted as day zero (day 0). Ewes were scheduled for slaughter on days of 12 (pre-implantation, *n* = 4; P12), 16 (implantation, *n* = 4; P16) or 22 (post-implantation, *n* = 4; P22) of gestation following mating, and on their corresponding days of 12 (*n* = 4, C12), 16 (*n* = 4, C16) or 22 (*n* = 4, C22) of the estrous cycle. To provide a similar effect of progesterone and to observe the only effect of pregnancy or embryo on plasma miRNA expression, cyclic ewes were exposed to a natural progesterone implant via intravaginal on day 13 of the cycle for days 16 and 22 groups, and progesterone implants were kept until the ewes were slaughtered. The presence of only one embryonic trophoblast was observed for 12, 16, and 22 days of pregnancy in the uterine lumen (Spencer et al., 2004; Bazer et al., 2012).

### Processing of blood sample and RNA isolation

We collected blood samples from ewes in tubes containing EDTA for plasma isolation just before the ewes were slaughtered. We centrifuged collected blood samples at 1,600×*g* for 13 min, and plasma was extracted and kept at − 80°C. Plasma was thawed at 20°C in the dry bath. Then, total cell-free RNA was extracted from 250 μl of plasma through miRCURY RNA Isolation Kit—Biofluids (Exiqon #300112 Vedbaek- Denmark) according to the manufacturer’s protocol. Plasma samples underwent on-column DNase to get rid of DNA contamination using the manufacturer’s protocols. Consequently, we eluted RNA samples using 40 μl RNase-free water. Tubes containing the miRNA were kept at −80°C until the miRNA array analysis.

### miRNA microarray

The profile of miRNAs from ovine plasma samples was investigated using the Affymetrix Microarray system with the GeneChip miRNA 4.0 Array (Affymetrix, United States) that is arranged to retrieve mature miRNA sequences in miRBase (20.0) (http://mirbase.org/ftp.shtml). Mature miRNA sequences of one hundred fifty from sheep are demonstrated in miRBase (20.0). A total of one 1) microgram of RNA was labeled with a FlashTagTM Biotin HSR RNA Labeling Kit (Affymetrix, United States). Following RNA labeling, through a GeneChip Hybridization Control Kit (Affymetrix, United States), microarray chips were hybridized with agitation at 60 rpm for 15 h. Then, the chip arrays were washed and subsequently stained through a Fluidics Station 450 (Affymetrix, Santa Clara, California, United States) with AGCC Fluidics Control Software. Fluorescence was detected from the array chip with an Affymetrix^®^ GeneChip Scanner 3000.

### Raw data preparation and statistical analysis

The signal of probes was generated as cell intensity files (*CEL files) computed using Affymetrix GeneChip Command Console software and analyzed in Transcriptome Analysis Console software. The intensity data of each chip was processed through the robust multi-array average (RMA) and identified above background (DABG) normalization with a default analysis setting of Affymetrix. Probe set summarization was performed through Median Polish. Probe values were generated as log2 transformed. Comparison between the cyclic and the pregnant samples was accomplished through fold-change with an independent *t*-test. One-way ANOVA was used to reveal statistically significant genes at the significance level of *p* ≤ 0.05. To demonstrate biologically relevant gene expression changes of each of cyclic and pregnant conditions, the standard approach was employed through a *p*-value (*p* ≤ 0.05) as the primary criterion followed by fold change (−1.5 ≥ FC ≥ 1.5) as the secondary criterion to select differentially expressed genes. Upon first analysis, criteria were relaxed to (−1.25 ≥ FC ≥ 1.25; *p* < 0.05) to identify all the family members of miRNAs of interest in all the ovine plasma samples.

### Target gene prediction of differentially expressed miRNAs

The target gene prediction of miRNAs in plasma samples was accomplished using the miRNAconsTarget online tool from sRNAtoolbox (http://bioinfo5.ugr.es/srnatoolbox), providing consensus target prediction. The given input data are developed on independent prediction from animal-based tools. TargetSpy, PITA (energy score < −15), and miRanda (pairing score >150 and an energy score < −15), making a total of three prediction algorithms. The common target genes predicted by all three tools were considered a potential miRNA target.

### Protein-protein interaction network construction and module analysis

The functional network association among target genes was established using the database of STRING (version 11.5, http://string-db.org), and eventually visualized in Cytoscape (version 3.9.0). The PPI network of target genes was transferred and subsequently assessed in Cytoscape. The functional modules were determined and shown by Molecular Complex Detection (MCODE), a plugin in Cytoscape for identifying intensively connected nodes in a current network. The module was set as follows: k-score = 2, cut-off degree = 2, max depth size = 100, MCODE score >5, and node score cut-off = 0.2 (Lou et al., 2019). The nodes included in the main modules are demonstrated as densely connected genes that show significant biological functions. CytoHubb CytoScape plugin was used to reveal important nodes by integrating topological calculations such as Maximal clique centrality (MCC), Maximum neighborhood component (MNC), Degree, Edge percolated component (EPC), and (EcCentricity) EC (Chin et al., 2014). The overlapping genes were ranked using the aforementioned five algorithms.

### Gene ontology and pathway enrichment analysis of target genes

The KEGG pathway and GO enrichment for the predicted target genes from the modules were analyzed using Cytoscape software with the ClueGO V2.5.7 plug-in (Bindea et al., 2009). The ClueGO plug-in generates functionally grouped GO annotation networks for many target genes. The GO categories were assigned to molecular function (MF), cellular component (CC), and biological process (BP). Two-sided hypergeometric tests set the *p*-value to 0.05, and multiple test corrections were performed using Bonferroni step-down adjustment. The threshold of the kappa score was adjusted to 0.7.

### RT-qPCR

Forward primer, universal reverse primer, and Stem-Loop primer sequences of two miRNAs to be confirmed by RT-qPCR (Supplementary Material S1). For the confirmation with RT-qPCR, firstly, a reverse transcription reaction was prepared using the First-Strand cDNA (1 μg RNA to cDNA) Synthesis Kit for RT-qPCR (USB, Cat no: 75780). Reverse transcriptase reaction conditions were as follows: 32 min at 16°C, 60 min at 44°C, 10 min at 95°C, and 5 min at 4°C. Following cDNA synthesis by reverse transcriptase reaction, RT-qPCR analyses of the samples were performed using VeriQuest Fast SYBR Green RT-qPCR Master Mix (USB, Cat no: 75690). RT-qPCR reaction was used as follows: Polymerase for 8 min at 95°C, then 45 cycles of denaturation for 25 s at 94°C, annealing for 42 s at 58°C, and extension for 50 s at 70°C. Log transformation of the data was performed according to the previously mentioned 2−ΔCt method (Livak and Schmittgen, 2001). Statistical analysis of values normalized to reference genes was calculated by Relative Expression Software Tool (REST 2009) (Pfaffl et al., 2002).

## Results

### miRNA profiles of ovine plasma in estrous cycle and early pregnancy

**FIGURE 1:**
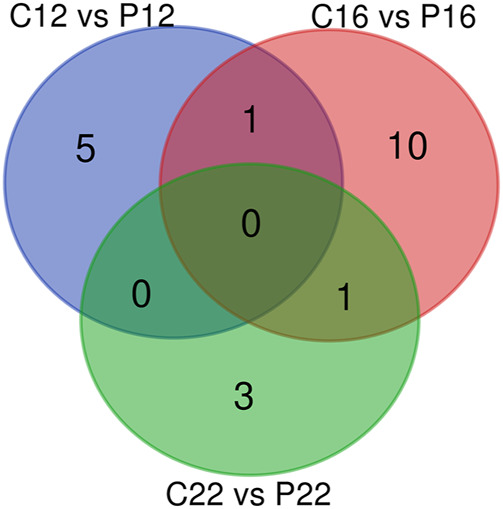
miRNA profiles in estrus cycle and pregnancy in ovine plasma. The Venn diagrams represent miRNA profiles between C12 and P12, C16 and P16, and C22 and P22.

Microarray results in plasma samples revealed a total of 183 miRNAs in all species between the estrous cycle and early pregnancy, while 60 were identified in ovine plasma samples (Supplementary Material S2). However, when respectively compared, there were 6, 12, and 4 statistically significant plasma-specific miRNAs between C12 vs. P12, C16 vs. P16, and C22 vs. P22, respectively. Among these, one was shared between C12 vs. P12, C16 vs. P16, C16 vs. P16 and C22 vs. P22, whereas 5, 10, and 3 miRNAs were unique to C12 vs. P12, C16 vs. P16, and C22 vs. P22, respectively (Figure 1).

### Differentially expressed miRNAs between cyclic and pregnant ovine plasma

**FIGURE 2:**
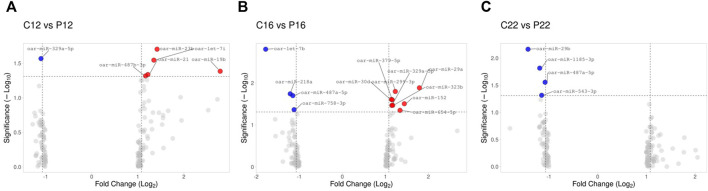
Illustration of volcano plot for differentially expressed miRNAs in estrus cycle and pregnancy in ovine plasma. The −log10 is set versus the log2 (fold change: cycle: pregnancy). **(A)** C12 vs. P12, **(B)** C16 vs. P16, and **(C)** C22 vs. P22. Color represents the fold change, red: up and blue: down.

A total of 22 differentially expressed ovine plasma miRNAs were determined (*p* < 0.05, difference of miRNAs greater than 1.25-fold change) when cyclic ovine plasma samples compared to pregnant ovine plasma samples. While five miRNAs (oar-miR-23b, oar-let-7i, oar-miR-19b, oar-miR-21, oar-miR-487b-3p were upregulated between C12 and P12, one of them (oar-miR-329a-5p) was downregulated (Figure 2A). Between C16 and P16, 8 miRNAs (oar-miR-29a, oar-miR-299-3p, oar-miR-30d, oar-miR-379-5p, oar-miR-152, oar-miR-323b, oar-miR-329a-5p, and oar-miR-654-5p) were upregulated while 4 miRNAs (oar-let-7b, oar-miR-218a, oar-miR-487a-5p, and oar-miR-758-3p) were downregulated (Figure 2B). In C22 vs. P22, miRNAs (oar-miR-29b, oar-miR-1185-3p, oar-miR-487a-5p, and oar-miR-543-3p) were downregulated (Figure 2C). The list of the 22 significant miRNAs from cyclic and pregnant groups with their fold change is depicted in Table 1. Furthermore, the detailed information on the differentially expressed ovine plasma miRNAs is demonstrated in Supplementary Material S2.

**TABLE 1:** The list of the differentially expressed miRNAs between cyclic days (C12, C16, and C22) and pregnant days (P12, P16, and P22).

Groups	Transcript ID	Accession	Fold Change	ANOVA *p*-value
C12 vs. P12	oar-miR-23b, oar-miR-329a-5p, oar-let-7i, oar-miR-19b, oar-miR-21, oar-miR-487b-3p	MIMAT0030049, MIMAT0019265, MIMAT0030026, MIMAT0030041, MIMAT0014966, MIMAT0019295	1.42, −1.1, 1.35, 2.79, 1.22, 1.17	0.019804, 0.027071, 0.028503, 0.04126, 0.046065, 0.048208
C16 vs. P16	oar-let-7b, oar-miR-29a, oar-miR-299-3p, oar-miR-218a, oar-miR-487a-5p, oar-miR-30d, oar-miR-379-5p, oar-miR-152, oar-miR-323b, oar-miR-329a-5p, oar-miR-758-3p, oar-miR-654-5p	MIMAT0014963, MIMAT0014967, MIMAT0019252, MIMAT0030045, MIMAT0019304, MIMAT0030059, MIMAT0019247, MIMAT0030035, MIMAT0019314, MIMAT0019265, MIMAT0019262, MIMAT0019282	-1.78, 1.79, 1.23, -1.21, −1.15, 1.14, 1.16, 1.44, 1.17, 1.15, -1.12, 1.34	0.001568, 0.013043, 0.015942, 0.01812, 0.020003, 0.0246, 0.025283, 0.031226, 0.033997, 0.034248, 0.043262, 0.044898
C22 vs. P22	oar-miR-29b, oar-miR-1185-3p, oar-miR-487a-5p, oar-miR-543-3p	MIMAT0030054, MIMAT0019289, MIMAT0019304, MIMAT0019272	−1.43, −1.19, −1.08, −1.15	0.006853, 0.015291, 0.027848, 0.048368

### Target prediction of selected microRNAs and PPI network construction

The target genes of the twenty-two miRNAs were predicted using the miRNAconsTarget online tool from sRNAtoolbox based on animal-based prediction with three algorithms (TargetSpy, PITA, and miRanda). Six differentially expressed miRNAs between C12 vs. P12 targeted 156 genes. Twelve miRNAs targeted 257 target genes between C16 and P16, whereas there were 108 in C22 and P22 by four differentially expressed miRNAs (Supplementary Material S3). Overlapping target genes from cyclic and pregnant were excluded, and then 326 were submitted to STRING online database. A PPI network including 328 nodes and 1,936 edges was directed employing the Cytoscape software (Supplementary Material S4).

### qPCR for miRNA expression analysis

**FIGURE 3:**
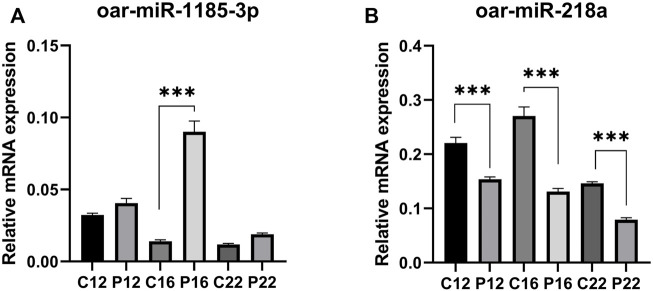
The relative abundance of selected miRNAs quantified by RT-qPCR were statistically significant. Expression of **(A)** oar-miR-218a and **(B)** oar-miR-1185-3p, between C12 vs P12, C16 vs P16, and C22 vs P22. Data are shown as relative abundance ± SEM, *p* < 0.05; (Estrous cyclic day 12: C12, Pregnant day 12: P12, Estrous cyclic day 16:C16, Pregnant day 16: P16, Estrous cyclic day 22:C22, Pregnant day 22: P22, indicates the group).

Among the miRNAs, oar-miR-1185-3p and oar-miR-218a were detected to be regulated in ovine plasma samples. The expression of oar-miR-1185-3p mRNA was similar between C12 and P12. It was found to be greater in P16 than in C16, while it did not change between C22 and P22 (Figure 3A). oar-miR-218a mRNA was found to be lower in pregnancy groups than in cyclic groups (C12 vs. P12, C16 vs. P16, and C22 vs. P22) identified (Figure 3B).

### Functional interaction network and module analysis of target genes

**FIGURE 4:**
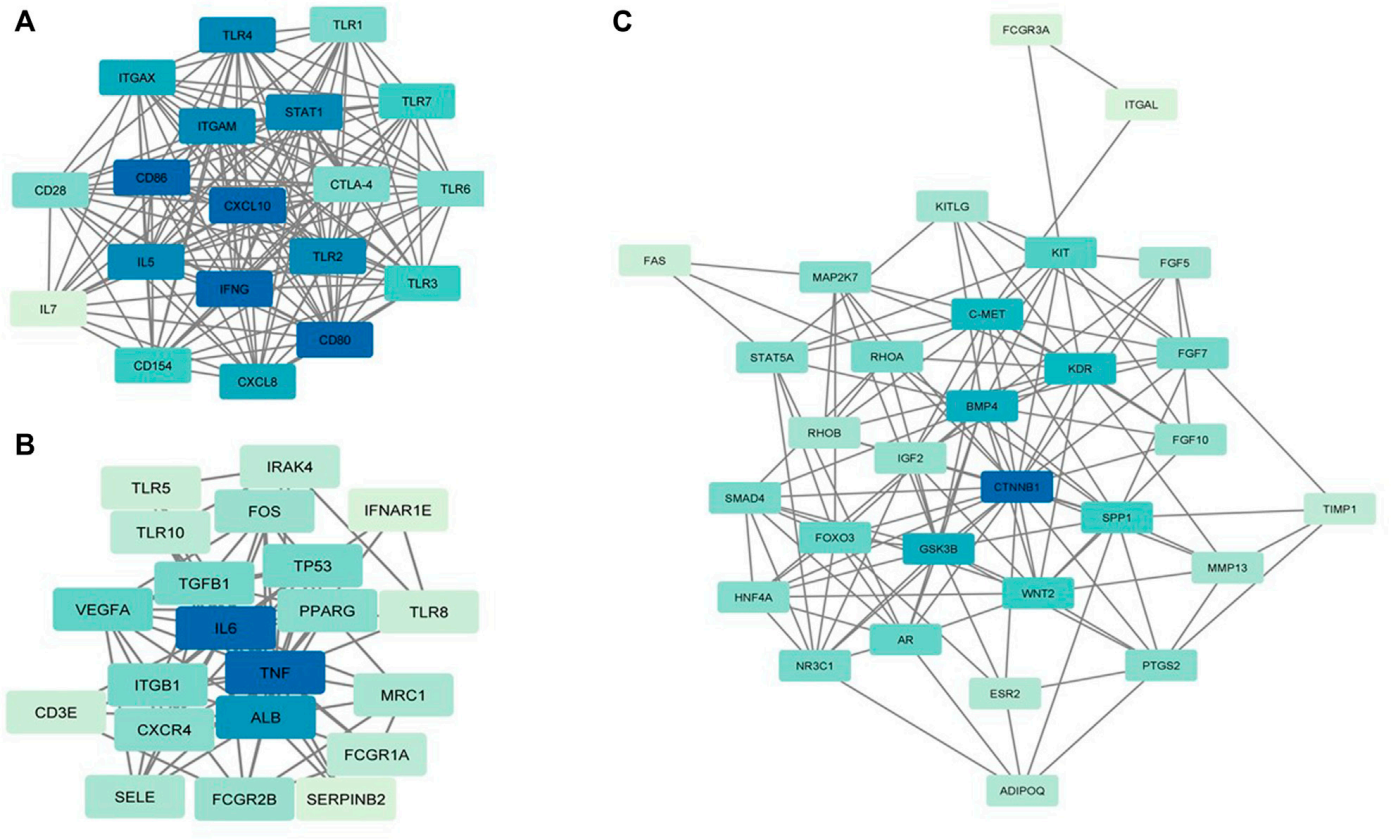
MCODE clustering of the top three clusters. **(A)** Module 1. **(B)** Module 2. **(C)** Module 3. In the modules, the nodes are colored in a continuous manner compliant with their |log2FC| values.

**FIGURE 5:**
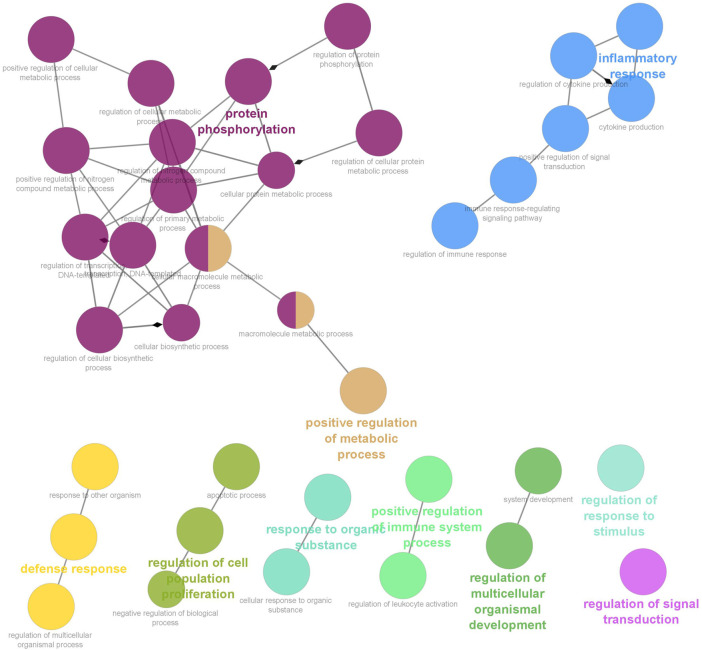
Functional interaction of network analysis of the target genes from module 1 between estrus cycle and pregnancy. GO terms are shown as nodes, the color depth of node shows distinct proportions of target genes. Nodes located in the same cluster are nominated as the same node color and size of nodes demonstrates the number of mapped genes in each GO term. Relationship between terms is shown by edges. Functionally associated groups partly overlap and are randomly colored.

**FIGURE 6:**
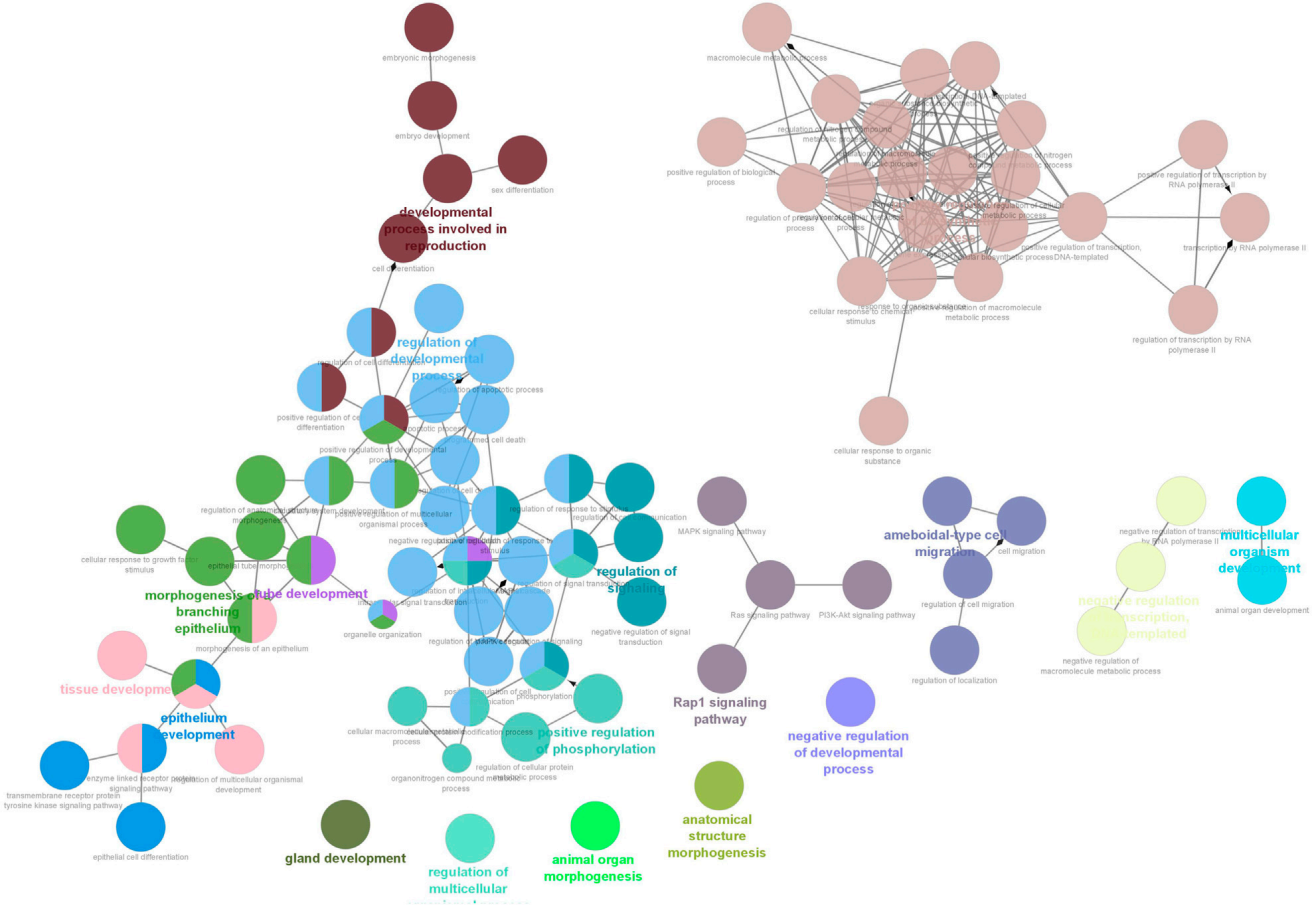
Functional interaction of network analysis of the target genes from module 2 between estrus cycle and pregnancy. GO terms are shown as nodes, the color depth of node shows distinct proportions of target genes. Nodes located in the same cluster are nominated as the same node color and size of nodes demonstrates the number of mapped genes in each GO term. Relationship between terms is shown by edges. Functionally associated groups partly overlap and are randomly colored.

**FIGURE 7:**
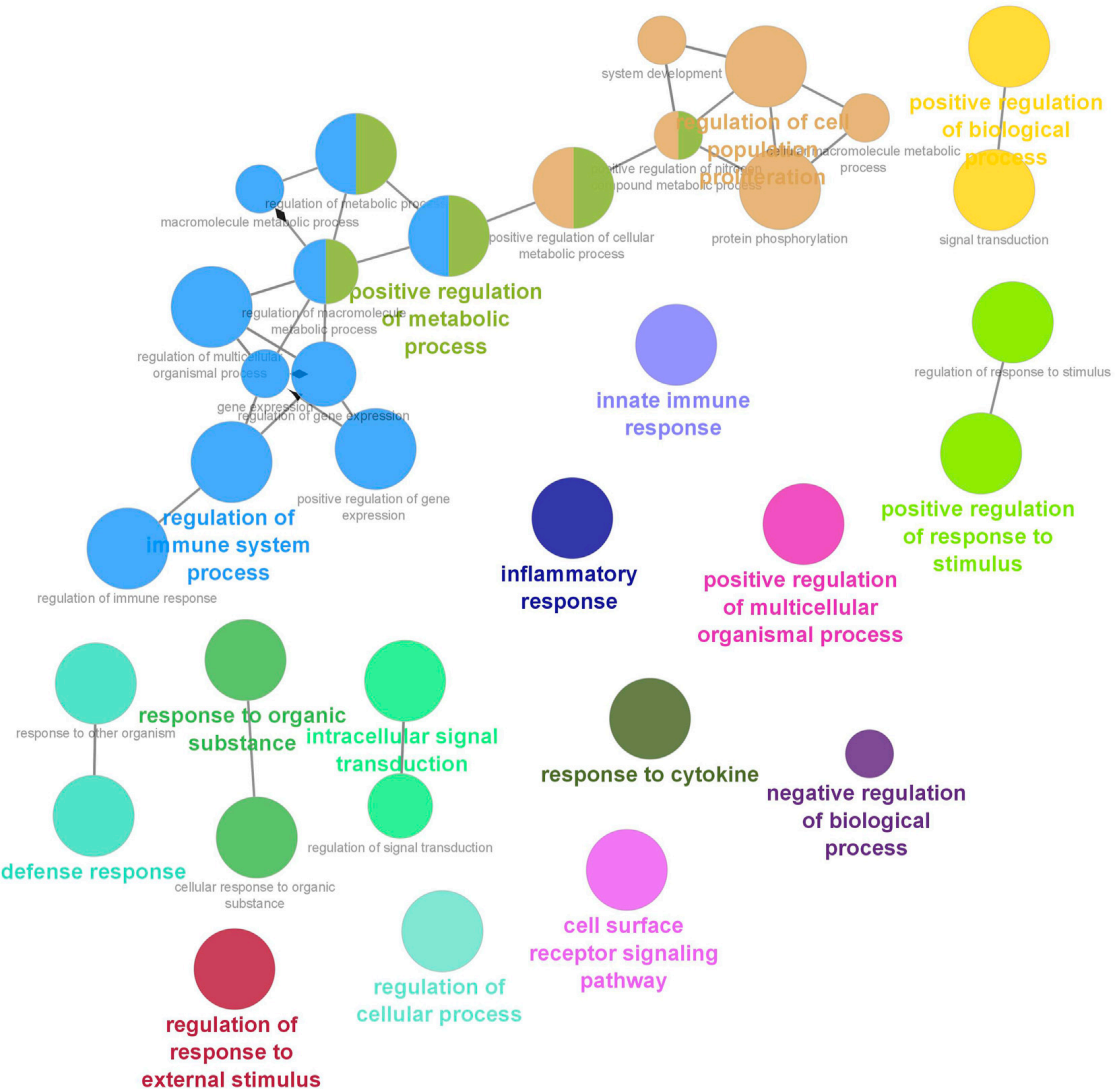
Functional interaction of network analysis of the target genes from module 3 between estrus cycle and pregnancy. GO terms are shown as nodes, the color depth of node shows distinct proportions of target genes. Nodes located in the same cluster are nominated as the same node color and size of nodes demonstrates the number of mapped genes in each GO term. Relationship between terms is shown by edges. Functionally associated groups partly overlap and are randomly colored.

Six clusters were attained from the PPI network following module analysis through the MCODE plugin of Cytoscape, and we sorted out three modules among all as hub modules constructed from MCODE scores (Figures 4A–C). Module 1 consisted of 19 nodes and 151 edges and had the highest MCODE score (16.778) of all modules (Figure 4A). Module 2 has 30 nodes and 131edges (Figure 4B) Supplementary Material S5, whereas module 3 contained 21 nodes and 84 edges (Figure 4C). All five classifications of methods within the CytoHubba plugin were accepted, and the highest ranked genes of each method (top 10) were shown (Table 2). Module 1 was enriched in (GO:0006468) protein phosphorylation, (GO:00069540) inflammatory response, (GO:0006952) defense response, (GO:0042127) regulation of cell population proliferation, (GO:0010033) response to organic substance, (GO:0009893) positive regulation of metabolic process, (GO:0002684) positive regulation of immune system process, (GO:2000026) regulation of multicellular organismal development, (GO:0009966) regulation of signal transduction (Figure 5). Module 2 was involved in (GO:0009790) embryo development, (GO:0003006) developmental process involved in reproduction, (GO:0048598) embryonic morphogenesis, (GO:0050793) regulation of developmental process, (GO:0035295) tube development, (GO:0060429) epithelium development, (GO:0061138) morphogenesis of a branching epithelium, (GO:0009891) positive regulation of the biosynthetic process (Figure 6). Although module 3 had common GO terms with module 1, it contained (GO:0006952) defense response, (GO:0050776) regulation of immune response, (GO:0045087) innate immune response, (GO:0010033) response to organic substance, (GO:0006954) inflammatory response, (GO:0051240) positive regulation of the multicellular organismal process (Figure 7) (Supplementary Material S6).

**TABLE 2:** Hub target genes of differentially expressed miRNAs from the modules. Overlapping hub protein symbols in the top 10, ranked methods were highlighted as bold. MCC: maximal clique centrality; MNC: maximum neighborhood component; Degree: node degree; EPC: edge percolated component; EC: EcCentricity.

	Node name	MCC	Node name	MNC	Node name	Degree	Node name	EPC	Node name	Ec Centricity
Module 1	CD80	1E+09	CD80	18	CD80	18	CD80	10.1	CD80	1
	CXCL10	1E+09	CXCL10	18	CXCL10	18	IFNG	10.0	IFNG	1
	CD86	1E+09	CD86	18	CD86	18	CXCL10	10.0	CXCL10	1
	IFNG	1E+09	IFNG	18	IFNG	18	CD86	9.9	CD86	1
	TLR2	1E+09	TLR2	17	TLR2	17	STAT1	9.8	STAT1	0.5
	TLR4	1E+09	TLR4	17	TLR4	17	TLR2	9.6	TLR2	0.5
	STAT1	1E+09	STAT1	17	STAT1	17	CXCL8	9.6	CXCL8	0.5
	TLR3	1E+09	ITGAM	17	ITGAM	17	TLR4	9.6	TLR4	0.5
	TLR1	1E+09	IL5	17	IL5	17	IL5	9.6	IL5	0.5
	TLR6	1E+09	CXCL8	16	CXCL8	16	ITGAX	9.5	ITGAX	0.5
Module 2	Node name	MCC	Node name	MNC	Node name	Degree	Node name	EPC	Node name	Ec Centricity
	CTNNB1	2,826	CTNNB1	22	CTNNB1	22	CTNNB1	10.73	CTNNB1	0.5
	GSK3B	2,298	GSK3B	15	GSK3B	15	GSK3B	9.52	C-MET	0.5
	AR	2,174	BMP4	15	BMP4	15	BMP4	9.49	GSK3B	0.33
	SMAD4	2,166	KDR	14	KDR	14	C-MET	9.2	BMP4	0.33
	HNF4A	2,160	C-MET	13	C-MET	14	KDR	9.06	KDR	0.33
	BMP4	1770	WNT2	12	WNT2	12	WNT2	8.77	WNT2	0.33
	FOXO3	1,464	SPP1	12	SPP1	12	SPP1	8.35	SPP1	0.33
	WNT2	834	AR	10	KIT	11	AR	8.06	AR	0.33
	NR3C1	734	FOXO3	10	AR	10	KIT	7.96	KIT	0.33
	KDR	516	KIT	10	FOXO3	10	FOXO3	7.88	FOXO3	0.33
Module 3	Node name	MCC	Node name	MNC	Node name	Degree	Node name	EPC	Node name	Ec Centricity
	TNF	11040	TNF	20	TNF	20	IL6	15.67	IL6	1
	IL6	11040	IL6	20	IL6	20	TNF	15.64	TNF	1
	ALB	11004	ALB	16	ALB	16	ALB	15.51	ALB	0.5
	VEGFA	10800	VEGFA	10	VEGFA	10	VEGFA	14.50	VEGFA	0.5
	TP53	10080	TP53	9	TP53	9	TGFB1	14.42	TGFB1	0.5
	TGFB1	10080	TGFB1	9	TGFB1	9	TP53	14.35	TP53	0.5
	ITGB1	5,784	ITGB1	9	ITGB1	9	ITGB1	14.18	ITGB1	0.5
	CXCR4	5,760	CXCR4	8	CXCR4	8	CXCR4	14.11	CXCR4	0.5
	PPARG	5,064	PPARG	8	PPARG	8	PPARG	13.78	PPARG	0.5
	FOS	5,040	FOS	7	FOS	7	FOS	13.35	FOS	0.5

## Discussion

In this current study, we carried out profiling of plasma miRNA expression to determine potential miRNAs involved in the critical stages of peri-implantation. The circulating miRNA expression profile was evaluated on days 12, 16, and 22 during pregnancy after mating and on their corresponding days of 12, 16, and 22 of the estrous cycle. We already know that the estrous cycle in ewes is 17 days and declining progesterone levels start on day 16 after mating. Therefore, in the C16 and C22 groups, an external supply of progesterone was added. As a result, we were able to compare the plasma miRNA profiles of cyclic and pregnant sheep without being affected by the fluctuating levels of progesterone. We may therefore hypothesize that whatever changes we detected in the levels of miRNA expression in ovine plasma in pregnant ewes were a direct result of the conceptus given that progesterone levels were similar across the cyclic and pregnant groups.

Circulating plasma miRNAs have been detected in several studies that indicated a significant number of miRNAs to reveal the importance of transcriptomics in reproductive biology (Hitit and Kurar, 2017; Zong et al., 2021). As such, many miRNAs originating from villous trophoblasts circulate through maternal blood within or linked with extracellular vesicles (Miura et al., 2010; Cameron et al., 2011; Kotlabova et al., 2011). It has been identified that 374 human placenta-specific miRNAs, of which 10 with the highest global expression were also reflected in plasma (Smith et al., 2021). In another exploratory study, 208 plasma-specific miRNAs were identified in cattle; of these miRNAs, sixteen were statistically different between non-pregnant and pregnant plasma samples (Ioannidis and Donadeu, 2016). However, this is the first study elucidating the identification of circulating miRNA profiles in ovine plasma during the estrous cycle and pregnancy. In the current study, we discovered a total of 60 ovine circulating miRNAs between cyclic and pregnant plasma, indicating remarkable outcomes for sheep reproductive biology. Among these, 22 were differentially expressed in microarray results upon pregnancy groups were compared with cyclic (Table 1). The results may indicate that circulating plasma miRNAs may function as potential biomarkers in the regulation of pregnancy during the estrous cycle and early pregnancy (Ioannidis and Donadeu, 2017; Lim et al., 2021).

Our study is noteworthy that the target prediction of selected miRNAs uncovered a great many GO terms that ovine circulating miRNAs in plasma might regulate biological and molecular control of gene expression during early pregnancy. Accordingly, we particularly ascertained critical GO terms that may be involved in early pregnancy, such as (GO:0006468) protein phosphorylation, (GO:00069540) inflammatory response, (GO:0006952) defense response, (GO:0042127) regulation of cell population proliferation, (GO:0009893 positive regulation of metabolic process, (GO:0010033) response to organic substance, (GO:0002684) positive regulation of immune system process, (GO:2000026) regulation of multicellular organismal development, (GO:0009966) regulation of signal transduction. This may illuminate that circulating miRNAs clarify molecular events during pregnancy recognition and embryo implantation because extracellular vesicles derived from the serum, trophectoderm, and uterine epithelia in pregnant ewes are implicated in intercellular communication, and microRNA-mediated pathways are involved in the key pathway in the pregnancy (Brooks et al., 2016; Ioannidis and Donadeu, 2017; Sun et al., 2021; Ono et al., 2022).

The potential of circulating miRNAs has been demonstrated, in many animals, to be identified in maternal circulation directly following initiation of implantation (Sun et al., 2021). Based upon this knowledge, for instance, elevated expression levels of circulating miRNAs (miR-23b) are shown to be expressed within either pregnant endometria (Krawczynski et al., 2015) or serum (Reliszko et al., 2017) on a gestational day 16 of pregnant pigs. In sheep serum, expression of miR-23b, miR-30d, and miR-379 was found to be at greater levels in exosomes obtained from the umbilical vein on gestational day of 90 (Cleys et al., 2014). However, in line with the expression of miR-23b in pregnancy, our study detected higher expression levels of oar-miR-23b as early as day 12 of pregnancy compared to C12. In addition, our unpublished data showed that oar-miR-23b tended to be higher in P16 than C16 in the endometrium. We also showed that oar-miR-30d and oar-miR-379-5p were higher in P16 than in C16. Because the capability to detect miRNAs for endometrium has enabled us to reveal pregnancy-related markers (Kose et al., 2022), it is intriguing to monitor common miRNAs for conceptuses and maternal bloodstream. As such, broadly conserved miRNAs (Let-7b) have been detected at different stages of pregnancy in trophoblast (Liu et al., 2015) and serum (Ioannidis and Donadeu, 2017). We demonstrated that let-7i and let-7b were differentially expressed between C12 vs. P12 and C16 vs. P16. Likewise, as identified higher expression of miR-29a in cattle maternal blood on day 39 of pregnancy (Sánchez et al., 2021), conserved miR-29 family members, oar-miR-29a and oar-miR-29b, were found to be expressed in C16 vs. P16 and C22 vs. P22 in ovine plasma, respectively.

The gene targets of differentially expressed miRNA are underrepresented in common categories (inflammatory response (GO:0006954), defense response (GO:0006952), and regulation of immune response (GO:0050776)) relating to immune responses in functional interaction of network analysis of module 1 and 3, meaning that the pregnancy-stimulated changes require both activation and suppression of immune functions through circulating miRNAs (Bidarimath et al., 2014) Among them, CD80 and CD86, costimulatory molecules, involved in signaling in T cell activation (Gool et al., 1996) and upregulated during early pregnancy in the cattle endometrium (Kamat et al., 2016). We showed that CD80 and CD86 was modulated by oar-miR-329a-5p, oar-miR-23b, oar-miR-654-5p, oar-miR-29b, and oar-miR-29a. In similar lines to our results, the miR-29 family has been implied in the regulation of the immune system (Liston et al., 2012) and detected as a potential biomarker for adverse pregnancy outcomes (Deng et al., 2020; Sørensen et al., 2021). As determined in our study, miR-19b and miR-29a were potential candidates for pregnancy detection in cows (Ono et al., 2022). miR-19b was also detected in ovine extracellular vesicles of cyclic and pregnant uterine luminal fluid on day 14 (Burns et al., 2014). In our unpublished data, as in ovine plasma, we found that miR-19b and miR-29a were differentially expressed in C16 vs. P16 and C22 vs. P22 in the ovine endometrium. In module 3, we illuminated that differentially expressed miRNAs targeted a couple of hub genes, which are interleukins, in other words, proinflammatory cytokines (TNF and IL6). oar-miR-19b between C12 and P12 targeted TNF, which plays an indispensable role in very early pregnancy. On the other hand, miRNAs may directly regulate the expression of interferon tau (IFNT)-stimulated genes (ISGs) in peripheral blood cells as they are secreted into the uterine cavity and partially enter the blood circulation directly. As such, CXCL10, classical type I IFN-stimulated genes, was among the hub gene in module 1 and targeted by oar-let-7b and oar-let-7i. In addition, we detected that miRNAs targeted toll-like receptors (TLRs) that are part of the innate immune system because they have roles in the stimulation of the acquired immune system (Akira and Takeda, 2004; Atli et al., 2018). TLR2 and TLR4 in module 1 enriched in (GO:0045087) innate immune response, targeted by oar-miR-30d, oar-miR-758-3p, and oar-miR-654-5p.

Module 2 showed that targets (ADIPOQ, BMP4, CTNNB1, FGF10, HNF4A, IGF2, SMAD4, WNT2) were related to functional GOs that may be involved in early pregnancy because it was enriched in (GO:0009790) embryo development and (GO:0048598) embryonic morphogenesis. CTNNB1 was one of hub genes targeted by oar-miR-218a, oar-miR-543-3p, oar-miR-1185-3p, and oar-miR-19b. Wnt/β-catenin signaling pathway has an important function in early embryogenesis (Liu et al., 2016), that *β*-catenin-regulated adhesion is essential for successful preimplantation of embryo development in mice (Messerschmidt et al., 2016). In line with gene expression, the Wnt/*β*-catenin signaling pathway requires strict control of miRNA regulation in embryo development (Stepicheva et al., 2015; Caronia-Brown et al., 2016). As discussed above, the development process occurring during embryonic development requires branching morphogenesis of organs and tissues. Our study revealed that target genes were enriched in (GO:0061138) morphogenesis of a branching epithelium and (GO:0060429) epithelium development. Of these, BMP4 was one of the early developmental genes expressed in the epithelium (Warburton et al., 2005; Geng et al., 2011) and reported post-transcriptionally regulated by miRNAs (Spruce et al., 2010; Battista et al., 2013). Accordingly, we showed that WNT2 and BMP4, other hub genes, were targeted by the same miRNAs (oar-miR-30d, oar-miR-29a). These results were corroborative with those circulating miRNAs that may play a functional role in early pregnancy to modulate embryonic development.

Although the inconsistency in the expression of oar-miR-1185-3p between microarray and RT-PCR emerged, miRNA expression of oar-miR-218a is consistent with microarray results between C16 and P16. This is consistent with reports that oar-miR-218a has been recently reported to be an early pregnancy marker of extracellular vesicles in ovine serum (Sun et al., 2021). Also, serum and plasma identification of miR-218 expression was associated with pregnancy disorder (Lu et al., 2017; Akgör et al., 2020). The limitations of this study were that due to the lack of compatible miRNA expression probes with ewe, we were unable to confirm more miRNA markers using RT-PCR.

## Conclusion

In our study, using microarray and qPCR profiling in ovine, we discovered for the first time changes in the levels of miRNAs in plasma during early pregnancy. Differently expressed miRNA profiles in the ovine plasma during the estrus cycle and early pregnancy indicate that miRNAs may function as promising candidates for diagnosis in early ovine pregnancy. More specifically, predicted target genes in modules showed different functional GO such as (GO:0003006) developmental process involved in reproduction, (GO:0009790) embryo development, and (GO:0002684) regulation of immune system process that circulating miRNAs may enable the identification of networks to reveal the molecular basis of early events during pregnancy in sheep.

## Data Availability

The datasets presented in this study can be found in online repositories. The names of the repository/repositories and accession number(s) can be found below: EMBL-EBI ArrayExpress, E-MTAB-11709.
