# Supplementary material for: Circulating miRNAs in maternal plasma as potential biomarkers of early pregnancy in sheep
Source: Front Genet. 2022 Aug 17;13:929477. doi: 10.3389/fgene.2022.929477 (PMC9428447; doi:10.3389/fgene.2022.929477)
Supplement: Supplementary file 1 [file Table1.DOCX]

**Table S1**. The list of miRNA primers used for gene expressions in RT-qPCR

| **Transcript ID** | **Sequences of forward primer, universal reverse and stem-loop primers** |
| --- | --- |
| oar-miR-218a | FP: 5'- gtttgggttgtgcttgatctaa- 3' |
|  | RP: 5'- gtgcagggtccgaggt- 3' |
|  | S-LP: 5'- gttggctctggtgcagggtccgaggtattcgcaccagagccaacacatgg- 3' |
| bta-miR-1185 | FP: 5'- gtggagaggataccctttg- 3' |
|  | RP: 5'- gtgcagggtccgaggt- 3' |
|  | S-LP: 5'- gttggctctggtgcagggtccgaggtattcgcaccagagccaacaacata- 3' |
