# Supplementary figures and images for: Circulating miRNAs in maternal plasma as potential biomarkers of early pregnancy in sheep
Source: Front Genet. 2022 Aug 17;13:929477. doi: 10.3389/fgene.2022.929477 (PMC9428447; doi:10.3389/fgene.2022.929477)

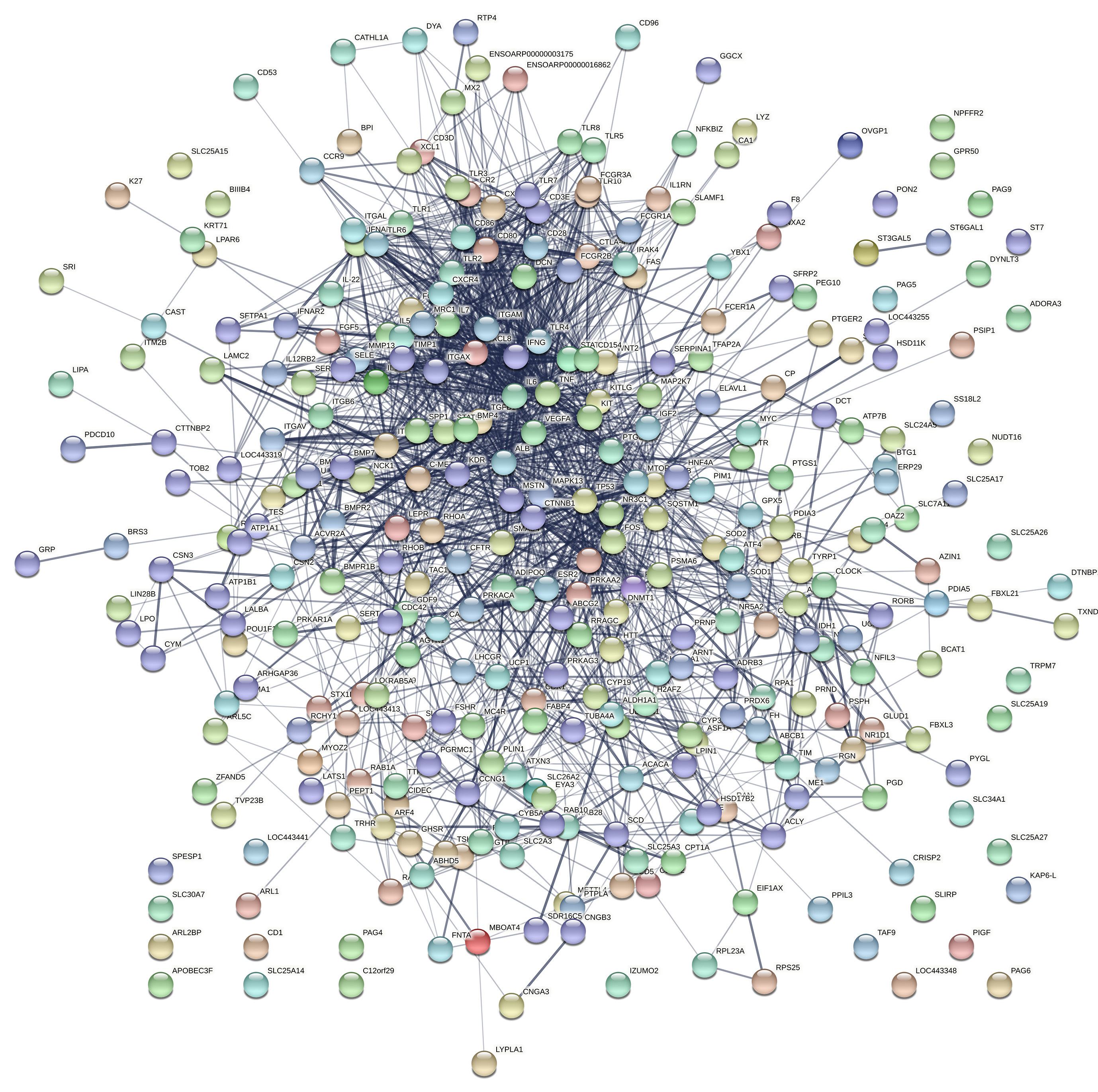

Supplement: Supplementary file 4 [file Image1.JPEG]
